# Emphysema early diagnosis using X-ray diffraction enhanced imaging at synchrotron light source

**DOI:** 10.1186/1475-925X-13-82

**Published:** 2014-06-21

**Authors:** Linan Dong, Jun Li, Wushuai Jian, Lu Zhang, Mingshu Wu, Hongli Shi, Shuqian Luo

**Affiliations:** 1 Department of Biomedical Engineering, Capital Medical University, Beijing 100069, China

**Keywords:** Pulmonary emphysema, Diffraction enhanced imaging, Multiple image radiography, Early diagnosis

## Abstract

**Background:**

Chronic obstructive pulmonary disease (COPD) is one of the leading causes of morbidity and mortality worldwide, and emphysema is a common component of COPD. Currently, it is very difficult to detect early stage emphysema using conventional radiographic imaging without contrast agents, because the change in X-ray attenuation is not detectable with absorption-based radiography. Compared with the absorption-based CT, phase contrast imaging has more advantages in soft tissue imaging, because of its high spatial resolution and contrast.

**Methods:**

In this article, we used diffraction enhanced imaging (DEI) method to get the images of early stage emphysematous and healthy samples, then extract X-ray absorption, refraction, and ultra-small-angle X-ray scattering (USAXS) information from DEI images using multiple image radiography (MIR). We combined the absorption image with the USAXS image by a scatter plot. The critical threshold in the scatter plot was calibrated using the linear discriminant function in the pattern recognition.

**Results:**

USAXS image was sensitive to the change of tissue micro-structure, it could show the lesions which were invisible in the absorption image. Combined with the absorption-based image, the USAXS information enabled better discrimination between healthy and emphysematous lung tissue in a mouse model. The false-color images demonstrated that our method was capable of classifying healthy and emphysematous tissues.

**Conclusion:**

Here we present USAXS images of early stage emphysematous and healthy samples, where the dependence of the USAXS signal on micro-structures of biomedical samples leads to improved diagnosis of emphysema in lung radiographs.

## Introduction

X-ray technology is widely used in medicine, biological and material research since Roentgen discovered X-ray in 1895. Now, imaging techniques used in clinical diagnosis mainly include X-ray fluoroscopy, X-ray photography and computed tomography (CT) imaging, etc., they derive contrast from difference in X-ray absorption. However, the differences in X-ray absorption coefficients of the structure in biological soft tissues are quite small, thus the contrast and spatial resolution of these techniques are quite low. When lesions were found by X-ray absorption-based radiography, the patient has missed the best period of treatment [1-3]. In the diagnosis of chronic obstructive pulmonary disease (COPD) also confronted with this problem.

COPD is one of the leading causes of morbidity and mortality worldwide [4], it is presumed that the mortality of COPD will rise to the third place in the world [5]. Emphysema is a common component of COPD, it is characterized by airflow limitation and excessive inflation which enlarge distal airspace and decrease the elasticity of pulmonary alveoli [4]. Chest X-ray is the conventional imaging methods for detection of emphysema. Due to changes in the lung tissue density of mild to moderate emphysema are quite small, the image of chest X-ray may be completely normal. In a study by Klein et al. [6], the sensitivity of chest radiography in the diagnosis of emphysema was 34%. Currently, early diagnosis of COPD and emphysema largely relies on spirometric lung function tests. However, because of the strong compensatory ability of lung, spirometric lung function tests may not be sensitive enough for measuring alterations in lung function at the early stages of emphysema [7]. A research [8] showed that the sensitivity of the spirometry in early diagnosis of the emphysema was about 80%. Histological examination is the most commonly method to observe micro-structure of biological tissues, it can observe the pathological change of early stage emphysema, but this method is invasive and not repeatable [9,10]. In our research, we identified mild emphysema successfully, and located lesion regions with false-color.

Lung is a kind of gas-bearing spongy tissue, compared with the phase contrast imaging, there is still a limit on the spatial resolution and contrast with the absorption-based CT [11]. In recent years, many studies have shown that X-ray phase contrast imaging of synchrotron radiation source can be used to observe the structure of the lung sample on the order of microns and conduce to lung disease diagnosis [11-13]. Now, the main phase contrast imaging methods include interferometry, diffraction enhanced imaging, in-line phase contrast X-ray imaging and grating-based X-ray imaging. Since Chapman proposed the diffraction enhanced imaging (DEI) method and put forward the calculation formulation of absorption image and refraction image, DEI has been widely used in various research fields. The DEI technique utilizes the analyzer crystal with an acceptance angle range of only a few micro-radians. The high angular sensitivity of the crystal allows the X-ray absorption, refraction, and ultra-small-angle X-ray scatter (USAXS) of the sample to be measured. The DEI information extraction [14,15] provided a way to present the absorption, refraction, and USAXS information individually. Those images possess higher contrast, and they can provide more detailed information of the sample.

## Materials and methods

### The principle of DEI

**Figure 1:**
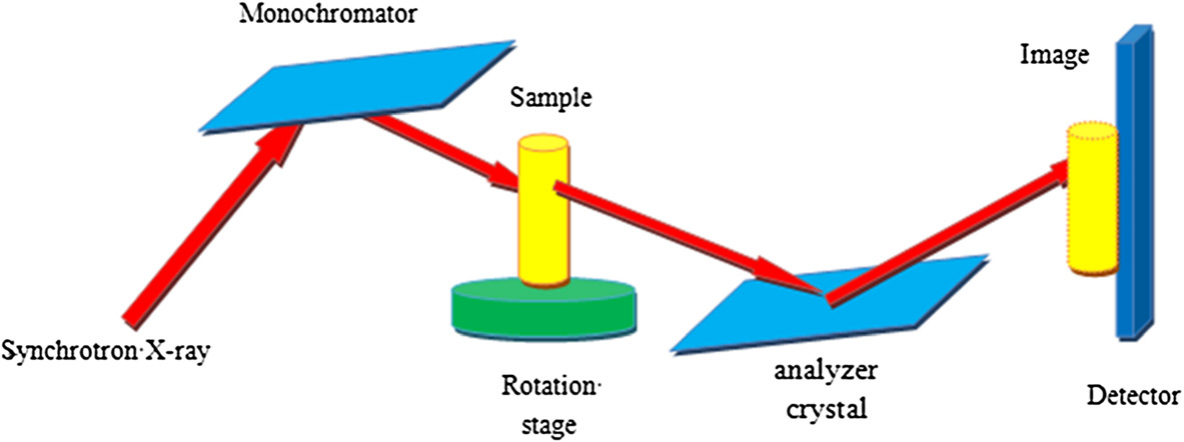
The DEI setup.

**Figure 2:**
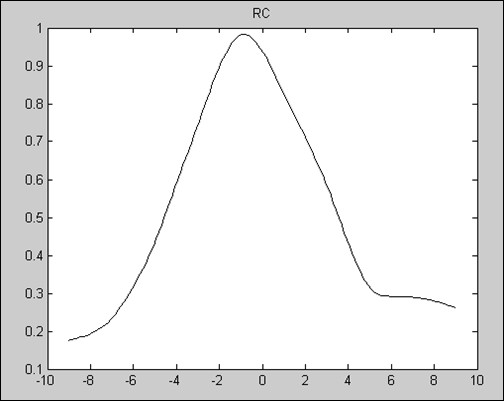
The rocking curve in the experiment.

The DEI setup is sketched in Figure 1, it is composed of two perfect crystals’ monochromator-analyzer system with a sample placed between them. The monochromator is used to generate a nearly monochromatic X-ray beam, the beam transmitted through the sample is incident upon the analyzer crystal, and the intensity of the X-ray beam reflected by the analyzer crystal is measured by a detector, which results in the formation of a DEI image [16]. When the X-ray beam transmits through a sample, the interactions between the X-ray and the sample include absorption, refraction, USAXS, and so on. When monochromatic crystal is fixed, and the analyzer crystal rotate in the meridian plan, we use the rocking curve to describe the reflectivity of the analyzer crystal as a function of the angle of analyzer crystal, as shown in Figure 2. According to Bragg’s diffraction law (2d * sin(θ ± Δθ) = n * (λ ± Δλ)), only the X-ray satisfying the Bragg condition of the analyzer crystal will be reflected onto the detector.

### Samples

Randomly selected six-wk-old Balb/c mice were used throughout this study. Mice had free access to water and rodent laboratory chow. The mice were anesthetized and applied papain through endotracheal instillation once a week (6 U/kg body weight), which continued four weeks. Then the animals were humanely sacrificed after ten days. The lung sample was prepared following the instillation-based inflation-fixation method established by Heitzman [17,18]. All experiments and procedures carried out on the animals were approved by Institutional Animal Care and Use Committee of Capital Medical University and the approved ID is AEEI-2014-049.

### Diffraction enhanced X-ray imaging

The experiments were conducted at the 4W1A beamline of Beijing Synchrotron Radiation Facility (BSRF). Two perfect crystals Si (111) were used as the monochromator and analyzer. The X-ray beam energy was set at 15Kev during the experiments. The detector had an VHR-16 M high resolution X-ray Imaging Camera system (Photonic Science Ltd.) with 4872*3248 pixels, and 7.4*7.4 μm^2^ per pixel, with a field of view of 36*24 mm^2^. Two series of DEI images were acquired in 23 positions of the RC, one with the sample and one without the sample, and they could be used to obtain absorption, refraction, and USAXS images. The samples were placed in a plastic container when acquire the DEI images.

### Image processing

In the study of information extraction method, Chapman et al. [16] presented the original information extraction method and produced images known as the apparent absorption image and refraction image using two images taken on either side of the RC of the analyzing crystal, in 1997. This method is simple to calculate, but the images acquired through this method are influenced by scattering, which reduces the spatial resolution [19]. Wernick et al. [20] proposed multiple image radiography (MIR) based on a statistical analysis. It can produce true absorption, refraction and USAXS images, respectively, and it is more robust to noise [20]. However, MIR increases the imaging time and X-ray radiation dose of the sample due to the need for more DEI images.

C.Hu et al. [15] made further explainations about MIR based on Wernick et al. [20]. In MIR, two series of DEI images are acquired in N (N>2) positions of the RC, one with the sample and one without the sample. In the following formulas [15], I_abs_, Δθ, $\sigma_{\theta }^{2},\;$ denotes the absorption, refraction and USAXS information, respectively, I_s_(θ_n_) (n =1,2,…,N ) denotes the intensity of the DEI image in the position θ_n_ of the RC with the sample. I_b_(θ_n_) (n=1,2,…,N) denotes the intensity of the DEI image in the position θ_n_ of the RC without the sample. The MIR absorption image is similar to a conventional radiograph, but exhibits greater contrast owing to the scattering rejection.

(1) $$I_{\mathrm{abs}}=-\ln\frac{\sum_{n=1}^{N}I_{s}\left(\theta_{n}\right)}{\sum_{n=1}^{N}I_{b}\left(\theta_{n}\right)}$$

(2) $$\Delta \theta =\theta_{s}-\theta_{b}=\frac{\sum_{n=1}^{N}I_{s}\left(\theta_{n}\right)\theta_{n}}{\sum_{n=1}^{N}I_{s}\left(\theta_{n}\right)}-\frac{\sum_{n=1}^{N}I_{b}\left(\theta_{n}\right)\theta_{n}}{\sum_{n=1}^{N}I_{b}\left(\theta_{n}\right)}$$

(3) $$\sigma_{\theta }^{2}=\frac{1}{\sum_{n=1}^{N}I_{s}\left(\theta_{n}\right)}\sum_{n=1}^{N}{\left(\theta_{n}-\theta_{s}\right)}^{2}I_{s}\left(\theta_{n}\right)-\frac{1}{\sum_{n=1}^{N}I_{b}\left(\theta_{n}\right)}\sum_{n=1}^{N}{\left(\theta_{n}-\theta_{b}\right)}^{2}I_{b}\left(\theta_{n}\right)$$

## Results

**Figure 3:**
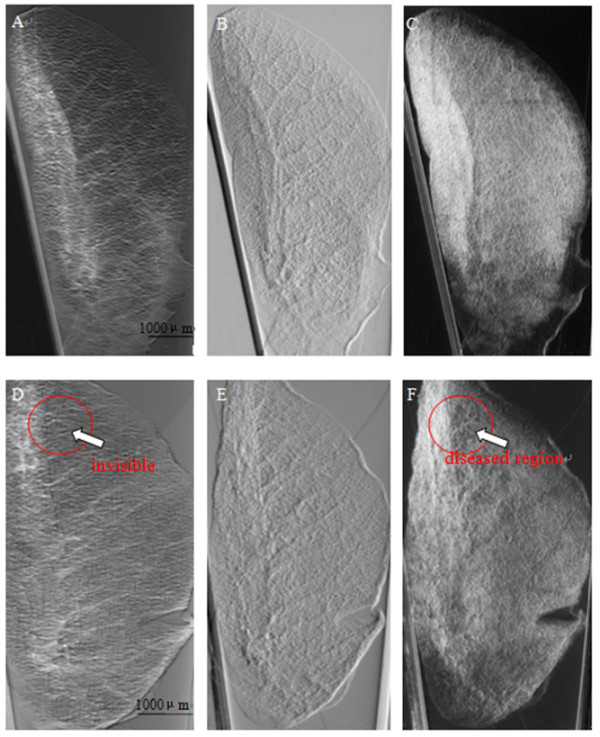
**Images of healthy and emphysematous samples. (A)**, **(B)**, **(C)** show the absorption, refraction and USAXS images of the healthy sample, respectively. **(D)**, **(E)**, **(F)** show the absorption, refraction and USAXS images of emphysema lung, respectively.

**Figure 4:**
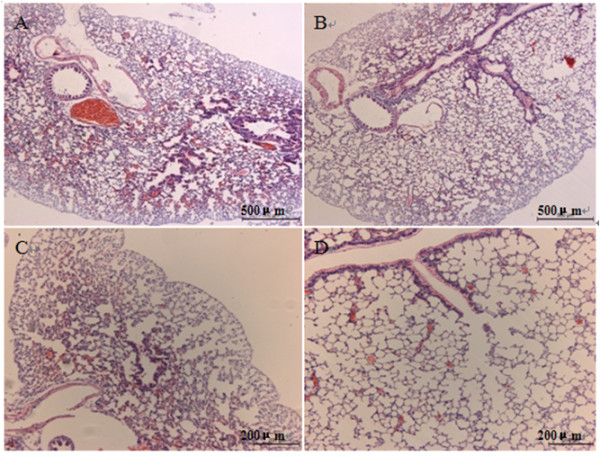
**Histological sections of healthy (A and C) and emphysematous lung sample (B and D).** Lung samples were embedded in paraffin and stained using a routine Mayer’s H & E staining protocol. Sections were scanned at various magnifications to create digital images. **(A and B)** 5× and **(C and D)**10 × magnification of the lung sections, respectively.

**Figure 5:**
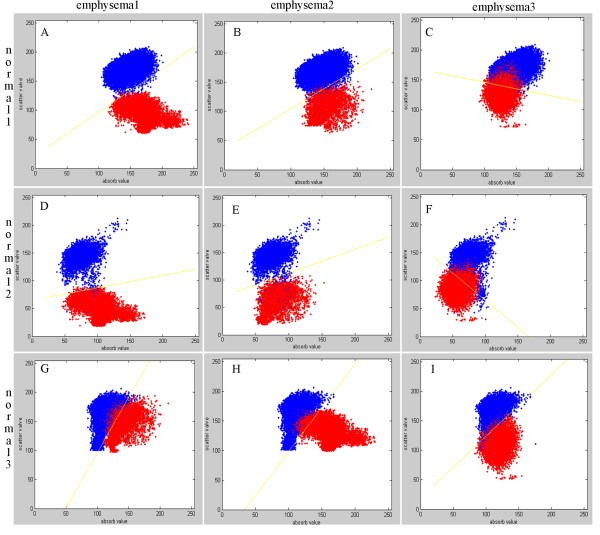
**Scatter plots of the absorption image and the USAXS image. (A)**,**(B)**,**(C)**,**(D)**,**(E)**,**(F)**,**(G)**,**(H)**,**(I)** are the scatter plots of the absorption image and the USAXS image of a healthy sample and a emphysematous sample. From this statistical analysis a clear distinction between emphysematous (red) and healthy (blue) lung tissue is possible. The straight line (yellow) produced by linear discriminant function can be regard as the boundary of the two groups.

**Figure 6:**
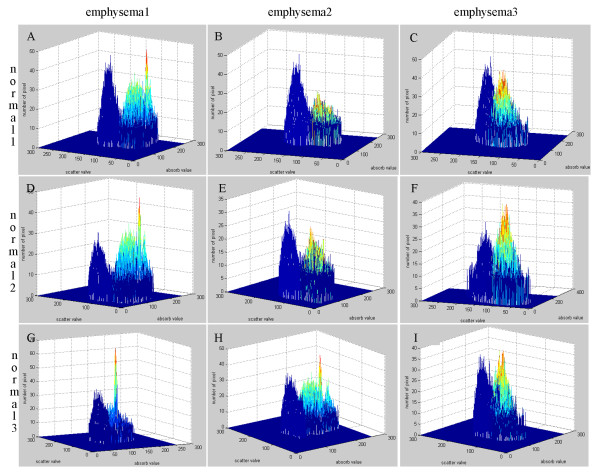
**Scatter plots of the absorption image and the USAXS image in three-dimensional.** The nine scatter plots in three-dimensional corresponding to the plots in Figure 5, respectively, the Z-axis presents the number of the pixels at XY plane's each position, the blue presents the healthy sample and the color presents the emphysematous sample.

**Figure 7:**
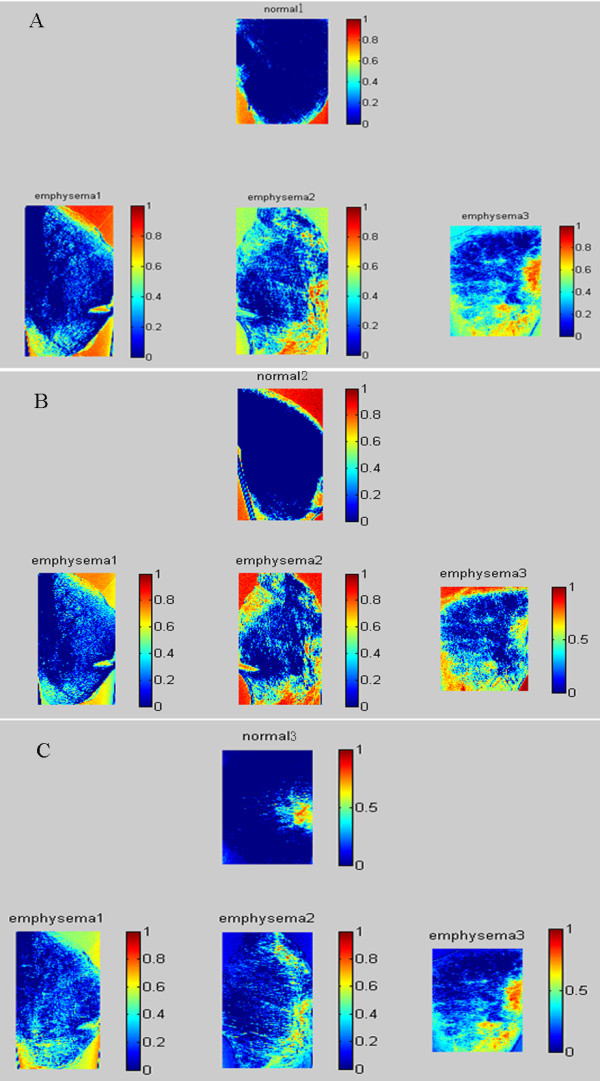
**Classification results of three emphysematous samples with each healthy sample.** Multimodal projections of three healthy lung samples (normal1, normal2, normal3) and three emphysematous lung samples (emphysema1, emphysema2, emphysema3). To visualize the apparent differentiation of healthy and emphysematous lungs from the scatter plot, we propose using the threshold values produced by linear discriminant analysis, and the threshold values are displayed in jet colors, where higher values (yellow, red) correspond to lower scattering and thus larger mean alveoli diameter. The (A),(B),(C) show that the diseased regions of the same emphysematous lung sample have the same varying tendency under different healthy sample.

Multiple projections of three emphysematous and three healthy mouse lung samples were acquired at the 4W1A beamline of Beijing Synchrotron Radiation Facility (BSRF). Then calculated the absorption, refraction and USAXS images using MIR. Figure 3(A), (B), (C), (D), (E), (F) show absorption, refraction and USAXS images of the healthy and emphysematous sample, respectively. Compared with the images of healthy mouse lung sample, the mean pixel value of emphysematous one is higher in absorption image but lower in USAXS image. The image texture of refraction image in emphysema lung is more irregular than it in the image of healthy sample. In a comparison of the absorption image, the USAXS image can be more clear to show the diseased region, as shown in the red circle in Figure 3.The change in alveoli diameter is well represented in the histological sections of healthy (Figure 4A and C) and emphysematous lung samples (Figure 4B and D), where a significant increase in mean alveolar diameter accompanied by the loss in lung tissue is apparent in the 10-fold magnification of emphysematous mouse lung tissue (Figure 4D).In order to get a better diagnosis of emphysema, we combined the absorption image with the USAXS image by a scatter plot. Figure 5 displayed the comparison results of each healthy and emphysematous lung sample by scatter plots respectively. The apparent discrimination of healthy and emphysematous lung tissues in the scatter plot can be visualized in the calculated projection images by linear discriminant function to select the pixels of emphysematous that show a large deviation from the healthy (blue) straight line. Compared to the deviation of the absorption images (X-axis) of the two groups, the deviation of the USAXS images (Y-axis) is more obvious, as shown in Figure 5. In other words, the USAXS image is more sensitive to diagnose the emphysema or even other pulmonary diseases. Figure 6 are the scatter plot in three-dimensional, the Z-axis presents the number of the pixels at the XY plane's each position, the blue presents the healthy sample and the color presents the emphysematous sample. The discrimination between the peaks of two kinds of samples is apparent, this indicates that the three-dimensional plot can provide more evidences for qualitative diagnosis.The main appearance of early stage emphysema is alveolar inflation, which changes the spatial frequency of tissue micro-structure. USAXS image is sensitive to the change of tissue micro-structure, it can show the lesions which are invisible in the absorption image. In scatter plots, lower pixel value means more serious alveolar inflation. According to those scatter plots, we compared all the emphysematous samples with each healthy sample to distinguish the disease level, as shown in Figure 7. The results displayed the changes of the micro-structure of the lung tissues in jet colors based on the boundary produced by linear discriminant analysis, where high values (yellow, red) correspond to lower scattering from the sample at comparable absorption properties, thus revealing larger mean alveoli diameter. The classification results in Figure 7 showed that the diseased regions of the same emphysematous lung sample had the same varying tendency under different healthy samples, which indicated the method we proposed is feasible.

## Discussion

Because of the low spatial resolution, CT results are limited mainly to structural analyses of larger airways. The change in X-ray attenuation caused by the pathological process of pulmonary emphysema is difficult to detect in early stage of the disease using conventional absorption-based radiography without contrast agents. Xenon ventilation CT using dual source and dual energy is a new technique for functional lung imaging which visualises lung ventilation and allows quantification of regional ventilation defects as well as gas trapping behind obstructed airways [21]. It can be used as a novel parameter to assess the severity of emphysema, but the defect is xenon may cause serious complication such as respiratory depression.

MR imaging of the lung is difficult because of its weak MR signal. Hyperpolarised helium-3 or xenon-129 are a new class of MR contrast agents ,they can be used to obtain high spatial resolution images of the lung airspaces. However, current capability to generate hyperpolarised gas for MR imaging is predominantly restricted to specialised centres in Europe and North America [21].

Currently, early diagnosis of COPD and emphysema largely relies on spirometric lung function tests. However, the tests are not so sensitive to the early stage emphysema. Compared with it, because of the high spatial resolution and no contrast agents, the DEI technique has more advantages in early emphysema diagnosis.

The scanning time in our experiments is a little longer than the time used in a conventional clinical chest radiograph, so a reduction of the number of scan steps is needed. It is observed that the rocking curve near the waist position can be similar to a straight line, where we can increase the scan interval angle and calculate the missing images using linear interpolation to reduce the scanning time and radiation dose.

## Conclusions

The small airways due to the specific anatomical and pathophysiological features do not usually cause clinical symptoms if the disease does not affect larger airways [22]. It seems that small airways and alveoli are the main point of airflow limitation and the attack occurs from the early stage in patients with COPD. Therefore, the role of the small airways and alveoli in the pathophysiology of obstructive lung diseases is important. However, no one method can currently be considered as a “gold standard” to assess the function of small airways.

In this paper, we utilized the MIR to extracted the absorption, refraction and USAXS images of the healthy and early stage emphysematous lung samples. A combination of absorption and USAXS information in a scatter-plot analysis presents improved emphysema diagnosis of mice lungs. We found that the strong dependence of USAXS information on the changes of micro-structure in lung tissue, especially in small airways and alveoli, plays an important role in early diagnosis of pulmonary emphysema.

In this study, the isolated mice lung samples were used in the experiments. In the future, with the development of technology and improvement of experimental equipments, research of human lung or other biological tissues in vivo will be conducted. Therefore, the early diagnosis based on the combination of multi-character of the DEI images will have extensive application prospects.

## Abbreviations

COPD: Chronic obstructive pulmonary disease; DEI: Diffraction enhanced imaging; USAXS: Ultra-small-angle X-ray scattering; MIR: Multiple image radiography; CT: Computed tomography; RC: Rocking curve; BSRF: Beijing synchrotron radiation facility; MR: Magnetic resonance.

## Competing interests

The authors declare that they have no competing interests.

## Authors' contributions

LD contributed to the formulation of the idea, performed experiments and drafted the manuscript, JL contributed to the conduction of experiments and participated in the development of the methods. WJ contributed to the conduction of experiments. LZ contributed to the conduction of experiments and reviewed the manuscript. MW contributed to the conduction of experiments. HS participated in the development of the method and reviewed the manuscript. SL is the Primary Investigator of the study under which this work was performed and participated in its design and reviewed the manuscript. All authors read and approved the final manuscript.
